# A sacrificial millipede altruistically protects its swarm using a drone blood enzyme, mandelonitrile oxidase

**DOI:** 10.1038/srep26998

**Published:** 2016-06-06

**Authors:** Yuko Ishida, Yasumasa Kuwahara, Mohammad Dadashipour, Atsutoshi Ina, Takuya Yamaguchi, Masashi Morita, Yayoi Ichiki, Yasuhisa Asano

**Affiliations:** 1 Biotechnology Research Center and Department of Biotechnology, Toyama Prefectural University, 5180 Kurokawa, Imizu, Toyama 939-0398, Japan; 2 Asano Active Enzyme Molecule Project, ERATO, JST, 5180 Kurokawa, Imizu, Toyama 939-0398, Japan

## Abstract

Soldiers of some eusocial insects exhibit an altruistic self-destructive defense behavior in emergency situations when attacked by large enemies. The swarm-forming invasive millipede, *Chamberlinius hualienensis*, which is not classified as eusocial animal, exudes irritant chemicals such as benzoyl cyanide as a defensive secretion. Although it has been thought that this defensive chemical was converted from mandelonitrile, identification of the biocatalyst has remained unidentified for 40 years. Here, we identify the novel blood enzyme, mandelonitrile oxidase (ChuaMOX), which stoichiometrically catalyzes oxygen consumption and synthesis of benzoyl cyanide and hydrogen peroxide from mandelonitrile. Interestingly the enzymatic activity is suppressed at a blood pH of 7, and the enzyme is segregated by membranes of defensive sacs from mandelonitrile which has a pH of 4.6, the optimum pH for ChuaMOX activity. In addition, strong body muscle contractions are necessary for *de novo* synthesis of benzoyl cyanide. We propose that, to protect its swarm, the sacrificial millipede also applies a self-destructive defense strategy—the endogenous rupturing of the defensive sacs to mix ChuaMOX and mandelonitrile at an optimum pH. Further study of defensive systems in primitive arthropods will pave the way to elucidate the evolution of altruistic defenses in the animal kingdom.

Swarm-forming animals have unique defense systems for protection. In eusocial insects that establish sophisticated castes, such as ants, for example, the ant soldiers use defensive behaviors that involve releasing defensive chemicals, biting with their mandibles, and stinging^1^. To address emergencies when attacked by large enemies, some ants and termites have evolved “Kamikaze” or altruistic self-destructive defense behavior as an instantaneous defense^2^,^3^,^4^.

On the other hand, the swarm-forming primitive arthropods, millipedes, usually individually use irritant chemicals to avoid attacks from omnivorous or carnivorous predators instead of establishing castes like the eusocial animals^5^,^6^. Mandelonitrile is a defensive chemical that is conserved among cyanogenic millipedes and is also used as a starting material to produce benzaldehyde, hydrogen cyanide, and benzoyl cyanide as defensive secretions. The aldoxime-nitrile pathway in the synthesis of mandelonitrile through phenylacetaldoxime and phenylacetonitrile is widely observed in bacteria, cyanogenic plants, and millipedes^7^,^8^,^9^,^10^.

The invasive polydesmid millipede, *Chamberlinius hualienensis* Wang, derives from Taiwan^11^. The animal usually forms a large swarm in cedar forests (see Supplementary Fig. S1) and has been expanding its range throughout southern Japan. When a cyanogenic millipede is attacked by a predator, it achieves a high blood pressure by forming a tight defensive spiral and can then expel benzoyl cyanide as a defensive chemical^5^,^6^,^12^,^13^. This chemical can disrupt the ant antennae functions, thus acting as an effective repellent. In addition, the reactive chemical causes irritation of the nose, eyes, and mouth of vertebrates such as birds, lizards, and humans^14^. It has been proposed that benzoyl cyanide is converted from mandelonitrile by dehydrogenation^5^. However, identification of mandelonitrile dehydrogenase has remained unidentified for 40 years.

Here, we identify the novel enzyme that catalyzes the synthesis of benzoyl cyanide from mandelonitrile. The enzyme is actually classified as an oxidase. We characterize its enzymatic activity, physico-chemical properties, and localization. Surprisingly, the substrate and the enzyme are not colocalized; they separately accumulate in defensive sacs and in blood respectively. Synthesis of benzoyl cyanide likely occurs by, endogenously rupturing the membranes of the defensive sacs using strong body muscle contractions during defensive behavior. We discuss this self-destructive defense of the millipede for protection of its swarm by synthesizing benzoyl cyanide from mandelonitrile through mandelonitrile oxidase.

## Results

### Identification, purification, and characterization of mandelonitrile oxidase from the invasive millipede

On the basis of a preliminary experiment using millipede homogenate as an enzyme source, we detected the production not only of benzoyl cyanide but also of hydrogen peroxide from mandelonitrile. To characterize the enzyme, we purified it using ion exchange and gel-filtration column chromatographies (see Supplementary Table S1) and analyzed its physico-chemical properties and activities.

**Figure 1:**
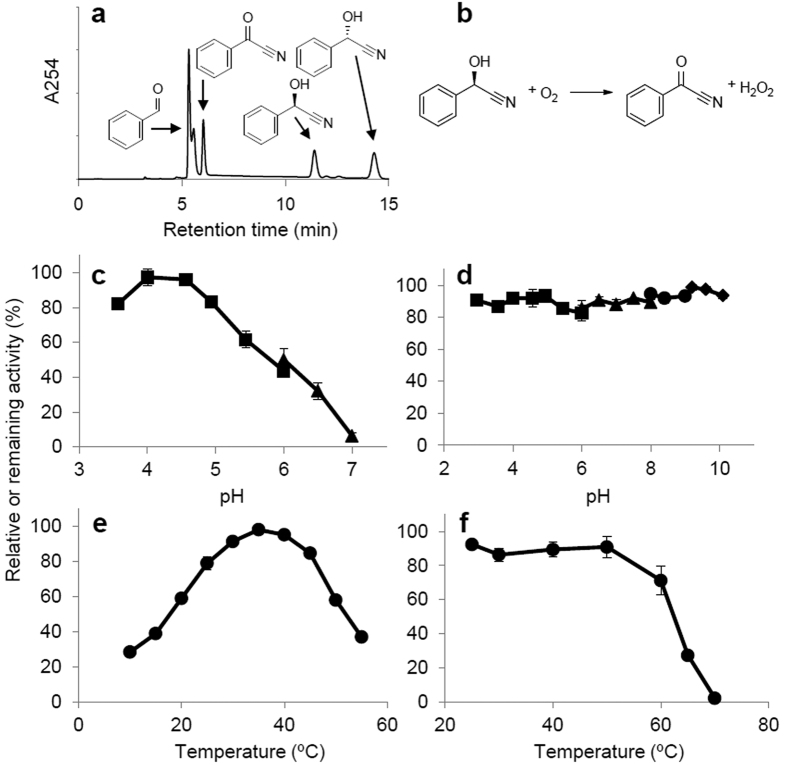
Characterization of the purified ChuaMOX. (**a**) Synthesis of benzoyl cyanide from racemic mandelonitrile. ChuaMOX is able to catalyze the synthesis of benzoyl cyanide at pH 5, and the retention times for benzaldehyde, benzoyl cyanide, (*R*)-mandelonitrile, and (*S*)-mandelonitrile were 5.4 min, 6.1 min, 11.4 min, and 14.3 min, respectively. Arrows indicate the peaks corresponding to benzaldehyde, benzoyl cyanide, and racemic mandelonitrile. (**b**) Reaction of ChuaMOX. (**c**) Optimum pH. (**d**) pH stability. (**e**) Optimum temperature. (**f**) Temperature stability. In panels (**c**–**f**), the highest mean value of the activity at pH 4 in panel **c** was defined as 100% to determine relative activity. Values are the means ± SD; n = 3. In panels (**c**,**d**), the symbols ◾, ▴, ⦁ and ◆ indicate citrate, phosphate, Tris-HCl, and glycine-sodium hydroxide buffers, respectively.

The purified enzyme was capable of synthesizing benzoyl cyanide from mandelonitrile *in vitro* (Fig. 1a; see Supplementary Fig. S2), the conditions (0.1 U of ChuaMOX, 1 μmol of mandelonitrile, and 100 μl of aqueous buffer) for which were estimated based on values from an animal extract and crude homogenate^15^. Placing 0.1 U and 0.01 U of the purified enzyme in 1 ml of 100 mM citrate buffer with pH 5 containing 500 nmol of (*R*)-mandelonitrile at 25 °C for 1 min consumed 95.6 nmol and 7.30 nmol of oxygen and produced 106 nmol and 10.2 nmol of benzoyl cyanide and 100 nmol and 10 nmol of hydrogen peroxide, respectively. These results indicate that the enzyme stoichiometrically consumes oxygen and converts (*R*)-mandelonitrile into benzoyl cyanide and hydrogen peroxide, (Fig. 1b). The results suggest that the enzyme should be newly classified as mandelonitrile:oxygen oxidoreductase (EC 1.1.3.-). Thus, we named this enzyme mandelonitrile oxidase (ChuaMOX).

The molecular mass of ChuaMOX was estimated to be 67,000 Da by SDS-PAGE analysis (see Supplementary Fig. S3a) and 65,000 Da by gel-filtration. Periodic acid-Schiff (PAS) staining determined that this enzyme was glycosylated (see Supplementary Fig. S3b). UV-visible scanning detected three absorption maxima at 278 nm, 404 nm, and 469 nm (see Supplementary Fig. S4). Thin layer chromatography (TLC) identified the prosthetic group as flavin adenine dinucleotide (FAD) (see Supplementary Table S2).

The values of *K*_m_ and *V*_max_ of ChuaMOX for racemic mandelonitrile were calculated as 2.1 mM and 151.5 U/mg, respectively. The optimum pH toward mandelonitrile lay within a range of pH 4 and pH 4.5, and the enzyme also showed 80% activity at pH 3.5 and pH 5. Enzymatic activity decreased in conditions approaching neutral pH, and it was lowest at a pH of 7 (Fig. 1c). After one hour of incubation at 25 °C, 80% of the ChuaMOX activity remained over a range of pH values between 3 and 10 (Fig. 1d). The optimum temperature was 35 °C, and 90% of the enzymatic activity was observed at 30 °C and 40 °C (Fig. 1e). After one hour of incubation at pH 8, 80% of the activity remained between 25 °C and 50 °C, and all activity was lost at 70 °C (Fig. 1f). These results suggest that ChuaMOX is a stable enzyme similar to hydroxynitrile lyase, which was previously isolated from *C. hualienensis* (ChuaHNL) and considered as a potential industrial biocatalyst for the synthesis of cyanohydrins^8^. The formation of hydrogen peroxide constituted 100%, 29%, and 22% of the ChuaMOX activity toward mandelonitrile, (*E*)-2-hydroxy-4-phenylbut-3-enenitrile, and 2-(3-bromophenyl)-2-hydroxyacetonitrile, respectively, indicating that this enzyme specifically reacts with mandelonitrile (Table 1). The enzyme required (*R*)-mandelonitrile as a substrate rather than (*S*)-mandelonitrile (see Supplementary Table S3). Adding 1 mM potassium cyanide, sodium azide, and 8-hydroxyquinoline resulted in 12.7%, 28.6%, and 56% relative activities of ChuaMOX, respectively, but adding 1 mM ferric chloride enhanced the activity to 124.6% (see Supplementary Table S4).

### Cloning of cDNA of ChuaMOX, prediction of its properties, and the enzyme and substrate localization

**Figure 2:**
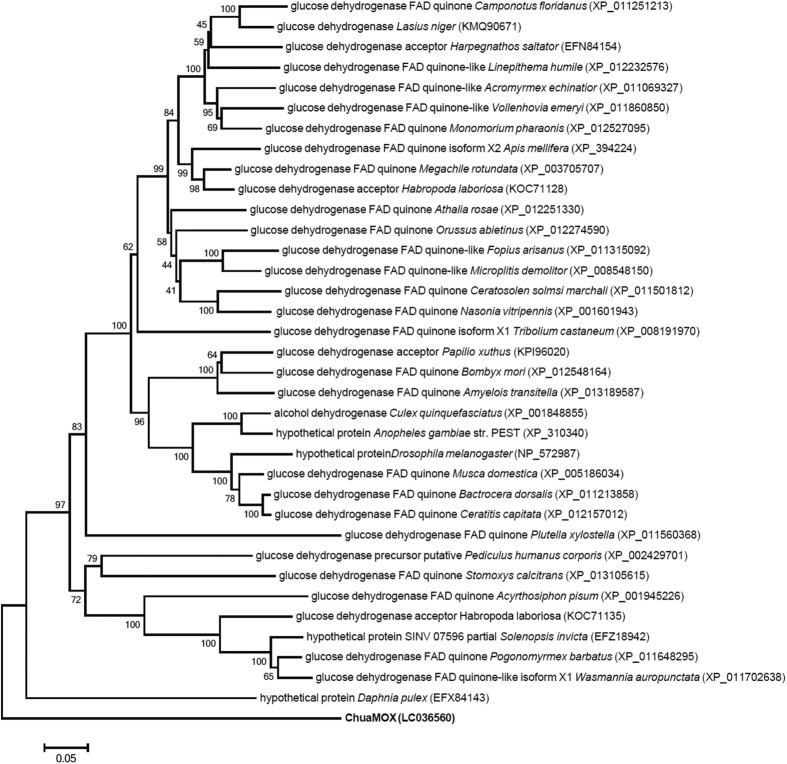
Phylogenetic analysis of ChuaMOX. ChuaMOX is shown in bold. The accession numbers of glucose dehydrogenases and alcohol dehydrogenases appear in parentheses. Bootstrap values were determined from 1,000 replications. A bar indicates a 5% divergence.

To further characterize ChuaMOX, we cloned its cDNA. Based on protein sequencing, cDNA was cloned using RNAseq data (BioProject ID, PRJDB3791) and gene-specific primers. The ORF consisted of 1,782 bp encoding 593 amino acid residues including a 17 amino acid-long signal peptide, and the deduced amino acid sequence also included 8 amino acid sequences determined by protein sequencing (accession number, LC036560; see Supplementary Fig. S5). The predicted mature protein had a molecular mass of 62,818 Da and an isoelectric point of 6.26. It also contained 6 predicted *N*-glycosylation sites (N57, N79, N94, N149, N406, and N491), and a FAD binding motif (VXGXGXXGXXXA)^16^ in the T22-S62 region (see Supplementary Fig. S5), which agree with the results of PAS staining, the UV-visible scanning, and the TLC analyses (see Supplementary Figs S3b and S4, and Table S2). BLASTP showed that it had a 51% amino acid identity to the predicted glucose dehydrogenase from the Florida carpenter ant, *Camponotus floridanus* (accession number, XP_011251213)^17^. However, phylogenetic analysis indicated that ChuaMOX did not belong to the cluster of glucose dehydrogenase and alcohol dehydrogenase (Fig. 2). The enzymatic activity not to react toward D-glucose (Table 1; see Supplementary Table S5) and the phylogenetic tree (Fig. 2) suggest that ChuaMOX is a distinctive enzyme stoichiometrically catalyzing oxygen consumption and the conversion of mandelonitrile into benzoyl cyanide and hydrogen peroxide and that it differs from glucose dehydrogenases and alcohol dehydrogenases.

**Figure 3:**
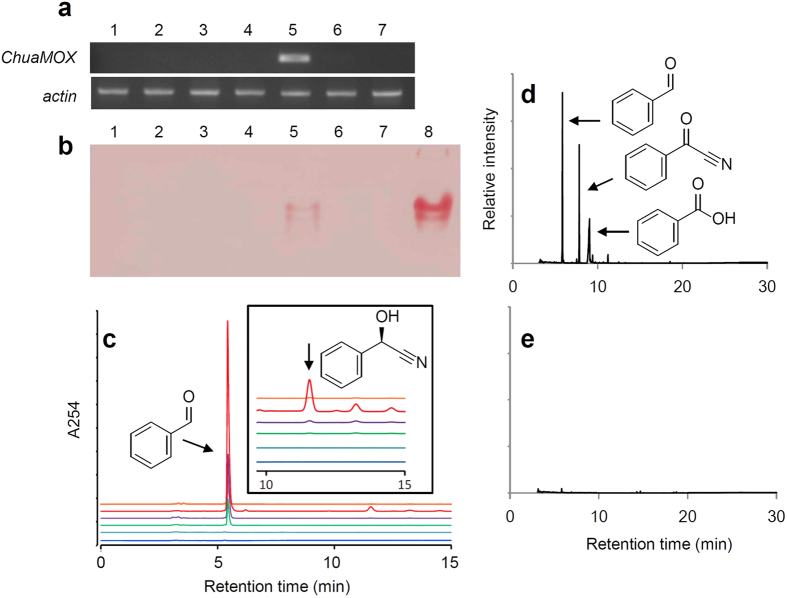
Localization of ChuaMOX and its substrate. (**a**) Expression of *ChuaMOX*. RT-PCR detects the gene expression in the paraterga, and *actin* expression is used as an internal control. (**b**) Localization of ChuaMOX. The enzyme activity was mainly detected in the blood by zymography. 1, Antenna. 2, Leg. 3, Head. 4, Integument. 5, Paraterga. 6, Fat body. 7, Gut. 8, Blood. (**c**) Localization of ChuaMOX substrate. (*R*)-Mandelonitrile is detected in the extract from the paraterga. Blue, Antenna. Light blue, Leg. Green, Head and tail. Purple, Integument. Red, Paraterga. Orange, Gut and fat body. Each extracted sample was analyzed using an HPLC equipped with a chiral column, and the inset shows a magnified view of the chromatogram for a retention time between 10 min and 15 min. Arrows indicate the peaks for benzaldehyde and (*R*)-mandelonitrile, respectively. Based on the calibration curves, the estimated amounts of benzaldehyde and (*R*)-mandelonitrile are 121 nmol and 93 nmol, respectively. (**d**) Whole body extract. The production of benzaldehyde, benzoyl cyanide, and benzoic acid detected by GC/MS analysis is indicated by arrows. (**e**) Blood extract. Cyanohydrins were not detected as substrates for ChuaMOX by GC/MS analysis.

*ChuaMOX* was expressed in the paraterga of the millipede (Fig. 3a), which houses the storage and reaction chambers related to the production of defensive secretions^6^. Because the storage and reaction chambers were fragile in various preparations of tissue sections, we could not detect cytological expression of ChuaMOX. Thus, we applied zymography to detect the enzyme activity of ChuaMOX from each experimental tissue. Intriguingly, most of the enzyme was detected in the blood (0.1 animal equivalent) (Fig. 3b), even though its pH of 7 is inappropriate for the expression of the enzymatic activity (Fig. 1c; see Supplementary Fig. S6). A scanty amount of the enzymatic activity was detected in the paraterga (1 animal equivalent), which may be derived from residual blood in the tissue (Fig. 3b). (*R*)-Mandelonitrile, a substrate of ChuaMOX, was detected from the paraterga by HPLC analysis but benzoyl cyanide was not (Fig. 3c). In addition, although defensive secretions such as benzaldehyde, benzoyl cyanide, and benzoic acid were observed in the whole-body extract (Fig. 3d), no candidate cyanohydrins for ChuaMOX were detected from the blood by gas chromatography-mass spectrometry (GC/MS) analysis (Fig. 3e). These results indicate that ChuaMOX is not only suppressed by the neutral pH of the blood, but it is also segregated from its substrate, (*R*)-mandelonitrile, by tissue membranes of the storage and reaction chambers. Furthermore, benzoyl cyanide is not stored in the defensive sacs but is newly synthesized during defensive behavior. In other words, the expression of benzoyl cyanide synthesis by the defense system is unusual in terms of the millipede’s physiology.

### Body muscle contractions are essential for mixing ChuaMOX and (*R*)-mandelonitrile, causing the production of benzoyl cyanide

**Figure 4:**
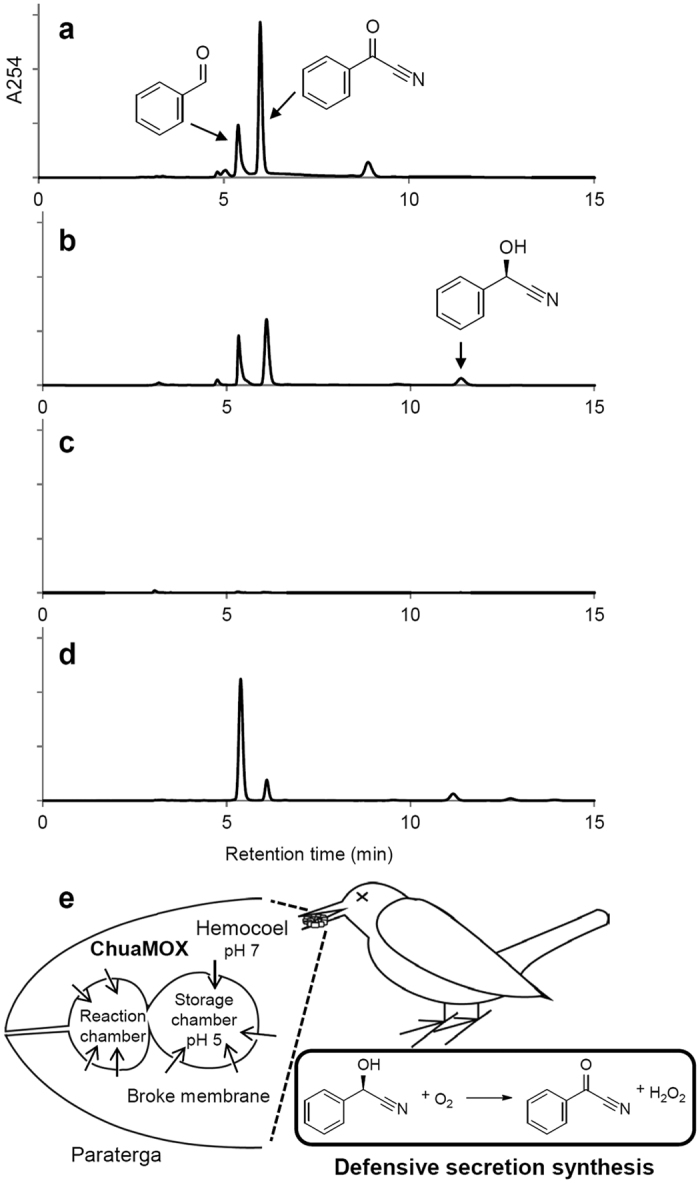
Synthesis of benzoyl cyanide as a defensive secretion *in vivo*. (**a**) Extract from a whole body. (**b**) Extract from a whole body after shaking. The arrows indicate peaks for benzaldehyde, benzoyl cyanide, and (*R*)-mandelonitrile, respectively. (**c**) Extract from a whole body after anesthetizing. The anesthetized animal is not able to release the defensive compounds including benzoyl cyanide. (**d**) Extraction from paraterga collected from the weakly anesthetized animal. The defensive sac in the paraterga was presumably ruptured by roughly snatching collection and residual blood and mandelonitrile were mixed. The isolated tissues synthesized benzoyl cyanide as non-anesthetized animals in (**a,b**). (**e**) Proposed defensive reaction of the millipede through ChuaMOX. A millipede caught by a predator strongly contracts its body muscles, and the behavior endogenously ruptures the membranes of the storage and reaction chambers. Drone blood enzyme, ChuaMOX, flows into the chambers from the hemocoel and is activated by the shift of pH from 7 to 4.6, which starts the synthesis of benzoyl cyanide from (*R*)-mandelonitrile.

To understand this enigma, we reexamined the synthesis of benzoyl cyanide in the millipede. The animal discharges defensive secretions, the major components of which are mandelonitrile, benzaldehyde, and benzoyl cyanide^12^,^13^. In the Japanese cedar forest, the animal is possibly attacked by large predators such as wild birds (jungle crow, *Corvus macrorhynchos*; Eurasian jay, *Garrulus glandarius*; dusky thrush, *Turdus eunomus*; and pale thrush, *Turdus pallidus*). These birds usually show behaviors of 1) swallowing in one gulp or 2) eating after teasing with a beak. Thus, we mimicked such attacks for 1) as a non-treated animal and 2) as a shaken animal, extracted the defensive secretion from each animal with an organic solvent (*n*-hexane:2-propanol with a volume ratio of 85:15). We then analyzed the extract using an HPLC equipped with a chiral column. The non-treated animal secreted benzaldehyde and benzoyl cyanide (Fig. 4a), while the shaken animal secreted benzaldehyde, benzoyl cyanide, and mandelonitrile (Fig. 4b). These results indicate that an active millipede is able to produce benzoyl cyanide in both conditions 1) and 2).

In the *n*-hexane/2-propanol extraction, we reproducibly observed that both experimental animals writhed in agony and exhibited strong body muscle contractions until death. It has been reported that a predator, the larva of the phengodid beetle, *Phengodes laticollis*, presumably injects a muscle relaxant into its millipede prey, *Floridobolus penneri*, before feeding to avoid the formation of a tight defensive spiral and the discharge of defensive secretions^18^. Thus, we hypothesized that inhibition of the body muscle contractions presumably prevents the production of benzoyl cyanide from the (*R*)-mandelonitrile. After anesthetizing using diethyl ether, the relaxed millipede did not display its defensive form or muscle contractions. HPLC analysis failed to detect not only benzaldehyde and mandelonitrile but also detected no benzoyl cyanide from the anesthetized animal (Fig. 4c).

Furthermore, we sought to synthesize benzoyl cyanide by roughly snatching the paraterga from the weakly anesthetized animals, which is expected to rupture defensive sac and mix residual blood containing ChuaMOX and mandelonitrile. HPLC analysis detected benzaldehyde, mandelonitrile, and benzoyl cyanide (Fig. 4d).

These results suggest that synthesis of benzoyl cyanide requires strong body muscle contractions in the tight defensive spiral position, presumably rupturing the defensive sacs and thus mixing ChuaMOX and mandelonitrile.

## Discussion

In this study, we isolate and localize ChuaMOX, which has remained unidentified for 40 years. It is stable in a wide range of pH and temperature conditions and is a monomeric glycosylated oxidase containing FAD as a prosthetic group. Furthermore, it stoichiometrically catalyzes the oxygen consumption and the synthesis of benzoyl cyanide and hydrogen peroxide from (*R*)-mandelonitrile and is different from glucose dehydrogenase and alcohol dehydrogenase. Thus, this enzyme is classified as mandelonitrile:oxygen oxidoreductase (EC 1.1.3.-). The enzymatic activity is expressed in a range of pH 3.5 to pH 5, whereas it is lowest at a blood pH of 7. The blood enzyme is segregated by membranes of the storage and reaction chambers from the substrate, (*R*)-mandelonitrile, in a condition of pH 4.6 (see Supplementary Fig. S6). *In vivo* experiments indicate that muscle contractions in the animal are essential for synthesis of benzoyl cyanide.

We propose the following molecular defense system for this invasive animal (Fig. 4e): when caught by a large predator, the millipede severely contracts its body muscle, which results in extremely high blood pressure. After endogenously rupturing the membranes of the storage and reaction chambers, the ChuaMOX in the blood immediately flows into these chambers due to the high blood pressure. The suppressed enzyme is activated by the shift of pH from 7 to 4.6 (see Supplementary Fig. S6), which starts the synthesis of benzoyl cyanide from (*R*)-mandelonitrile. Finally, the benzoyl cyanide is released from the sacrificed body, acting as a toxic exudate.

These defensive chemicals provide protection to both the animal under attack and its swarm. In some cases, the continuous release of the defensive chemicals from the prey contributes to the protection of its undamaged swarm from the large predator. Among eusocial insects, the carpenter ant, *Camponotus cylindricus* complex, also shows, endogenously rupturing its mandibular glands by body muscle contraction to exude defensive secretions of polyacetate-derived aromatics, aliphatic hydrocarbons, and alcohols from the mandibular gland^2^. Soldiers of the termite, *Globitermes sulphureus*, contract their body muscle, rupture the frontal gland membrane, and exude the defensive secretions^3^. The invasive millipede, *C. hualienensis*, is a larger arthropod than ants and termites. Its size (3.5 cm) makes it an appropriate pray for wild birds. Although it is not a eusocial animal with sophisticated castes, it also shows rupturing the defensive sacs, which consequently plays a role in protecting its swarm. In the field observation during millipede collection in 2013–2015, dusky thrushes approached the gutter filled with the millipedes. However, the wild birds showed just hopping several times toward the gutter, never attacked the swarm, and flew away all the time. Presumably they already learned that the millipedes were not eatable because of its defense system. Thus, the habitat of this millipede may be expanding in Japan yearly.

Insects and millipedes diverged into Chelicerata and Myriapoda 666 million years ago^19^. The basal lineages of ants and termites diverged approximately 105–110 million years ago and 130 million years ago, respectively^20^, and each developed eusocial forms. Self-destructive altruistic defenses have evolved independently in each eusocial form in response to various enemies^4^. Although this defensive strategy has been considered as a system unique to social animal, the invasive primitive millipede, which has not evolved eusociality, is also equipped with, rupturing the defensive sacs to protect its swarm. Further study on the defense systems in primitive arthropods will pave the way to delineate the establishment of altruistic defenses in the animal kingdom.

## Materials and Methods

### Millipede

Invasive millipedes of *C. hualienensis* [Polydesmida: Paradoxomatidae] were collected in Japanese cedar forests in southern Japan in 2013–2015 and shipped to our lab. The colony was maintained by supplying it with sliced sweet potatoes and water at 25 °C and 70–90% relative humidity. Alternatively, sampled animals were frozen with dry ice and stored at −80 °C until use.

### Measurement of enzymatic activity

The activity of ChuaMOX was measured by monitoring the rate of hydrogen peroxide formation using 2,2′-azinobis(3-ethylbenzothiazoline-6-sulfonic acid ammonium salt) (ABTS) (Dojindo, Kumamoto, Japan)^21^. The following reaction solution (1 ml) was prepared: 100 mM citrate buffer, pH 5, 0.1 mM ABTS, 5 mM racemic mandelonitrile (Sigma-Aldrich, St. Louis, MO, USA), and 150 mU horseradish peroxidase (Wako Pure Chemical Industries, Osaka, Japan). Following 5 min of preincubation, the enzyme solution was added to the reaction solution and mixed gently. The formation of hydrogen peroxide was measured by monitoring its absorbance at 405 nm and 25 °C for 1 min using a spectrophotometer (Evolution 201 UV-visible Spectrophotometer, Thermo Fisher Scientific, Waltham, MA, USA). Each point represents the mean value of three independent experiments, and one unit of activity is defined as the amount of the enzyme needed to catalyze the production of 1 μmol of hydrogen peroxide for 1 min.

### Purification of ChuaMOX from the invasive millipede

Frozen millipedes were thawed at room temperature, and the gut was removed using fine forceps under a microscope. The bodies without guts were homogenized in 10 mM Tris-HCl, pH 8, and centrifuged to remove debris.

All purification steps were carried out at 4 °C. The crude extract was loaded onto DEAE Sepharose FF (GE Healthcare, Little Chalfont, UK) and eluted with 10 mM Tris-HCl, pH 8, containing 50 mM sodium chloride. The diluted positive fraction was loaded onto Q Sepharose FF (GE Healthcare) and eluted with 10 mM Tris-HCl, pH 8, containing 25 mM sodium chloride. The concentrated sample was loaded onto Superdex 200 10/300 GL (GE Healthcare) and eluted with 20 mM Tris-HCl, pH 8, containing 150 mM sodium chloride at a flow rate of 0.5 ml/min. The purified enzyme is stable in 20 mM Tris-HCl, pH 8 at 4 °C for 6 months.

The protein concentration was determined using a protein assay kit (Bio-Rad Laboratories, Hercules, CA, USA) with bovine serum albumin as the standard protein. To estimate the molecular mass of the mandelonitrile oxidase, 10% SDS-PAGE and gel filtration were used.

### UV and visible spectral analysis

The absorption spectrum of the ChuaMOX (1 mg/ml in 10 mM Tris-HCl, pH 8) was recorded using a spectrophotometer (Evolution 201 UV-visible Spectrophotometer, Thermo Fisher Scientific).

### Thin layer chromatography analysis

Thin layer chromatography (TLC) analysis was followed using the methodology of the previous publication^22^.

### Detection of sugar molecules in ChuaMOX

After separation on 10% SDS-PAGE, the sugar molecules in ChuaMOX were detected by the periodic acid-Schiff (PAS) method using a Pierce Glycoprotein Staining Kit (Thermo Fisher Scientific).

### Characterization of ChuaMOX

The chemicals (see Supplementary Table S5) were purchased from various suppliers (Sigma-Aldrich, Dojindo, Alfa Aesar, and Tokyo Chemical Industries) or synthesized as described below and used as test substrates, inhibitors, and metal salts. Nitriles were prepared from their corresponding aldehydes by way of bisulfite adducts according to Young *et al.*^23^.

To determine optimum pH and pH stability, 100 mM of citrate buffer of pH 3.0–6.0, phosphate buffer of pH 6.0–8.0, Tris-HCl of pH 8.0–9.0, and glycine-sodium hydroxide of pH 9.0–10.0 were used. Enzyme activity was measured for a range of 10 °C to 70 °C to determine the optimum temperature and temperature stability. A 1-hour preincubation without substrate was used in the assays for pH stability and temperature stability and the other procedures were carried out as described above.

### Synthesis of benzoyl cyanide using the purified ChuaMOX

Benzoyl cyanide, (*R*)- and (*S*)-mandelonitrile, and benzaldehyde were used in known amounts of authentic chemicals to estimate retention time and preparation of calibration curves (see Supplementary Fig. S2). Because a millipede contains 1 μmol of (*R*)-mandelonitrile and 0.1 U of ChuaMOX in 100 μl of blood^15^ according to the calibration curve and the purification table, the same amounts of mandelonitrile and ChuaMOX were mixed in 100 μl of 100 mM of a pH 5 citrate buffer and incubated at 25 °C for 1 min. The product was analyzed using an HPLC equipped with a chiral column (CHIRALCEL OJ-H column: particle size, 5 μm; 4.6 mm i.d. × 250 mm; Daicel, Osaka, Japan)^8^.

### Measurement of oxygen consumption

Oxygen consumption in the enzyme reaction (nmol/ml/min) was monitored using a S1 Clark type polarographic electrode^24^ connected with an Oxygraph Plus oxygen electrode system (Hansatech Instruments, Norfolk, UK).

### Zymography

A millipede was completely anesthetized on ice, and blood was collected from small wounds. The paraterga were collected by fine forceps under a microscope. After immersing the body in a phosphate-buffered saline (PBS), other tissues were collected. Each tissue was homogenized in 10 mM Tris-HCl, pH 8. Protein extract equivalent to 1 animal of antenna, leg, head, integument, paraterga, and gut; 0.25 animal equivalent of fat body; and 0.1 animal equivalent of blood were loaded and separated on 10% native PAGE. Zymography was performed as follows: the gel was developed in 50 mM citrate buffer, pH 5 containing 0.6 mM 2-(4-indophenyl)-3-(4-nitrophenyl)-5-phenyl-*2H*-tetrazolim chloride (INT) (Dojindo Laboratories), 0.33 mM 1-methoxy-5-methylphenazinium methylsulfate (1-methoxy PMS) (Dojindo Laboratories), and 10 mM racemic mandelonitrile (Sigma-Aldrich).

### Detection of benzoyl cyanide as a defensive secretion from the millipede and the paraterga

Each millipede was treated with one of the following treatment: no treatment (control), shaking for 2 min, or anesthesia using diethyl ether. After each treatment, each animal was soaked in organic solvent (*n*-hexane:2-propanol with a volume ration of 85:15) for 3 min, and the extract was analyzed using an HPLC equipped with a chiral column^8^. On the other hand, the paraterga was collected from the weakly anesthetized animals by roughly snatching using a fine forceps to rupture the defensive sac and mix mandelonitrile and blood containing ChuaMOX.

### Gas chromatography-mass spectrometric analysis

A millipede or 10 μl of blood were transferred into a glass vial and immersed in *n*-hexane (5 ml) for 3 min at room temperature. The *n*-hexane extract (4 μl portion, each) was analyzed using a gas chromatography-mass spectrometer (GC/MS) (7890A GC System coupled with a 5975C inert XL EI/CI MSD with a Triple-Axis Detector operated at 70 eV; Agilent Technologies, Santa Clara, CA, USA) equipped with an HP-5 ms capillary column (0.25 mm i.d. × 30 m, 0.25 μm film thickness; Agilent Technologies) according to the previous publication^13^.

### Protein sequencing

After the separation of the purified ChuaMOX on 10% SDS-PAGE, the protein was visualized by Coomassie Brilliant Blue staining. The protein band was excised and treated with trypsin (sequencing-grade modified trypsin; Promega, Madison, WI, USA) following the previously reported methods^25^,^26^. The digested peptides were separated using a nanoUPLC equipped with a trap column (nanoACQUITY UPLC Symmetry C18 Trap Column, 100 Å, 5 μm, 180 μm x 20 mm, Waters, Milford, MA, USA) and a reverse phase capillary column (ACUITY UPLC Peptide CSH C18 nanoACQUITY Column 10 K psi, 130 Å, 1.7 μm, 75 μm × 200 mm, Waters). The molecular mass was determined with an electrospray ionization/quadrupole time-of-flight mass spectrometer (nanoESI-SYNAPT G2-Si mass spectrometry; Waters). The MS/MS spectra were processed using the Biolynx software suite (Waters).

### Transcriptome analysis

Total RNA was extracted from the animals without guts using a TRIzol Reagent (Thermo Fisher Scientific). After treatment with DNase I, the total RNA was re-purified using the RNeasy Mini Kit (Qiagen, Valencia, CA, USA), and the RNA samples were frozen with dry ice and shipped to Hokkaido System Science (Sapporo, Japan). After quality verification of the total RNA using an Agilent 2100 Bioanalyzer (Agilent Technologies), the cDNA library was constructed. To reduce the highly abundant transcripts before pyrosequencing, the library was normalized using the TRIMMER DIRECT cDNA normalization kit (Evrogen, Moscow, Russia) and pyrosequencing, including library construction, was performed at Hokkaido System Science. In brief, the raw reads sequenced using a GS FLX + system (Roche 454 Company, Branford, CT, USA) were cleaned by removing the adaptor sequence and unknown or low quality bases. *De novo* transcriptome assembly was performed using a GS De Novo Assembler v2.8 (Roche Applied Bioscience, Penzberg, Germany) with its default settings, and the assembled sequences were deposited in our local BLAST server. The *C. hualienensis* sequencing and assembly were summarized in Supplementary Table S6. The raw reads of sequence data was deposited in the DDBJ Sequence Read Archive (BioProject ID, PRJDB3791).

### cDNA cloning

A partial cDNA sequence encoding 5 amino acid sequences determined using a quadruple time-of-flight mass spectrometer was obtained from an in-house EST data base. Total RNA was isolated from the paraterga of *C. hualienensis* using a TRIzol Reagent (Thermo Fisher Scientific), and cDNA was synthesized using a SMART RACE cDNA Amplification Kit (Takara Bio, Kusatsu, Japan), a 5′-Full RACE Core Set (Takara Bio), and a GeneRacer Kit (Thermo Fisher Scientific). PCR was carried out using KOD plus neo (Toyobo, Osaka, Japan) and the gene-specific primers (see Supplementary Table S7), and the amplicons were sequenced using a DNA sequencer (3500 Genetic analyzer, Thermo Fisher Scientific). The full-length cDNA sequence was determined using 21 independent clones to avoid PCR-derived sequence errors, and the DNA and amino acid sequences of ChuaMOX were analyzed using GENETYX ver. 11 and ATGC (Genetyx, Tokyo, Japan), PeptideMass^27^, NetNGlyc 1.0 Server^28^, Cofactory^29^, and MEGA6^30^.

### RT-PCR

Each experimental tissue was collected as described above and immediately homogenized in a TRIzol Reagent (Thermo Fisher Scientific) after the addition of glycogen (Thermo Fisher Scientific). cDNA was synthesized using a SMART RACE cDNA Amplification Kit (Takara Bio) and SuperScript II (Thermo Fisher Scientific) as a reverse transcriptase. The gene-specific primers, ChuaMOX-1 and ChuaMOX-2 (see Supplementary Table S7), were used for RT-PCR. The expression of *actin* was detected as an internal control^31^.

### Measurement of the pH of blood and secretion from the ozopore

The pH of the solutions was estimated using pH indicator papers (Macherey-Nagel, Düren, Germany) according to previous publications^13^,^14^.

## Additional Information

**How to cite this article**: Ishida, Y. *et al.* A sacrificial millipede altruistically protects its swarm using a drone blood enzyme, mandelonitrile oxidase. *Sci. Rep.* **6**, 26998; doi: 10.1038/srep26998 (2016).

## Supplementary Material

Supplementary Information

## Figures and Tables

**Table 1:**
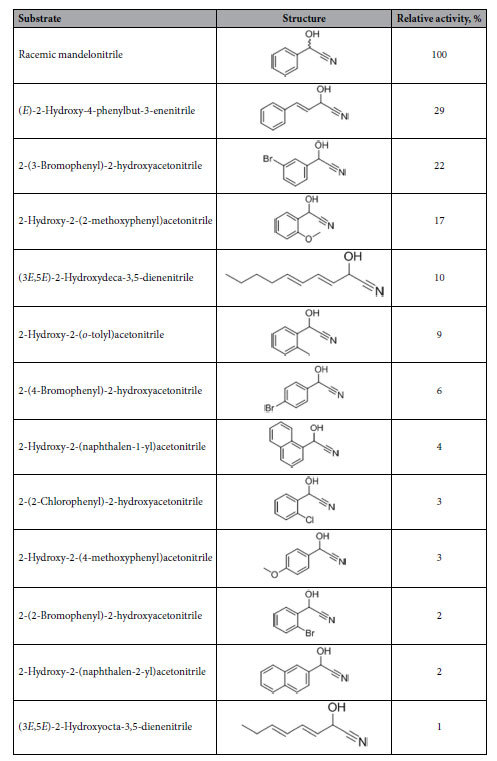
Substrate specificity of ChuaMOX.

ChuaMOX activity toward test substrates was detected under standard assay condition using ATBS. The relative activity was expressed as a percentage of the enzyme toward mandelonitrile (0.1 U/ml). The enzyme activity toward other substrates listed in Table S5 was not detected.
