# Supplementary material for: A sacrificial millipede altruistically protects its swarm using a drone blood enzyme, mandelonitrile oxidase
Source: Sci Rep. 2016 Jun 6;6:26998. doi: 10.1038/srep26998 (PMC4893617; doi:10.1038/srep26998)
Supplement: Supplementary Information [file srep26998-s1.pdf]

Title: A sacrificial millipede altruistically protects its swarm using a drone blood enzyme, mandelonitrile oxidase

Authors: Yuko Ishida[a,b], Yasumasa Kuwahara[a,b], Mohammad Dadashipour[a,b], Atsutoshi Ina[a,b], Takuya Yamaguchi[a,b], Masashi Morita[a,b], Yayoi Ichiki[a,b], and Yasuhisa Asano[a,b, 1]Supplementary Materials

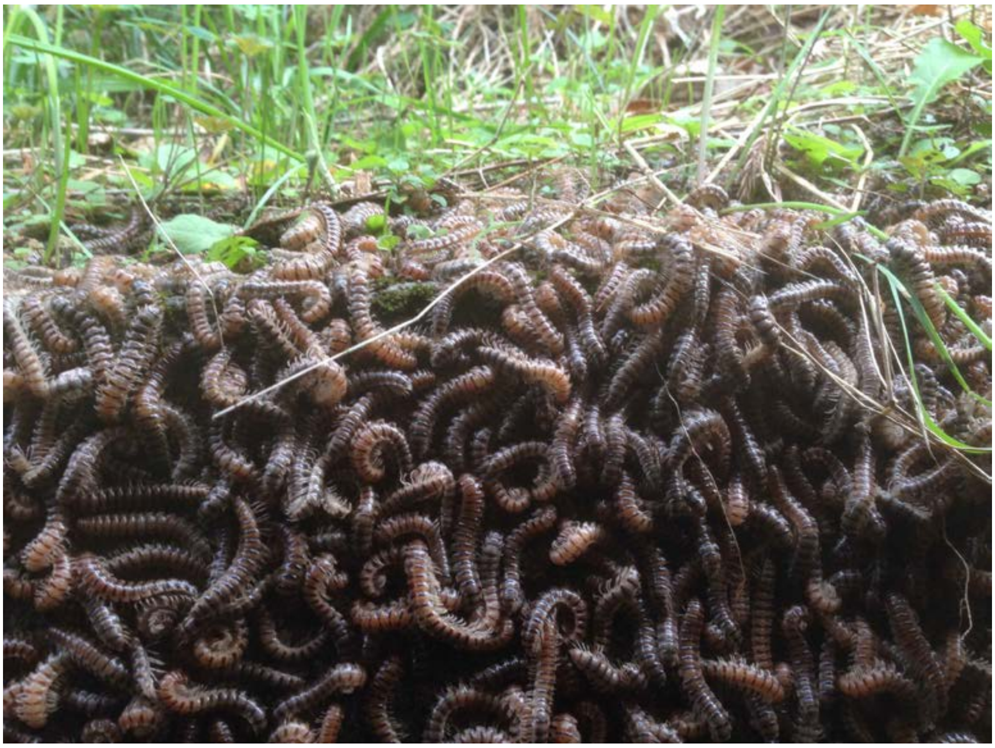

Fig. S1. A swarm of the invasive millipede, *C. hualienensis*, in a cedar forest.

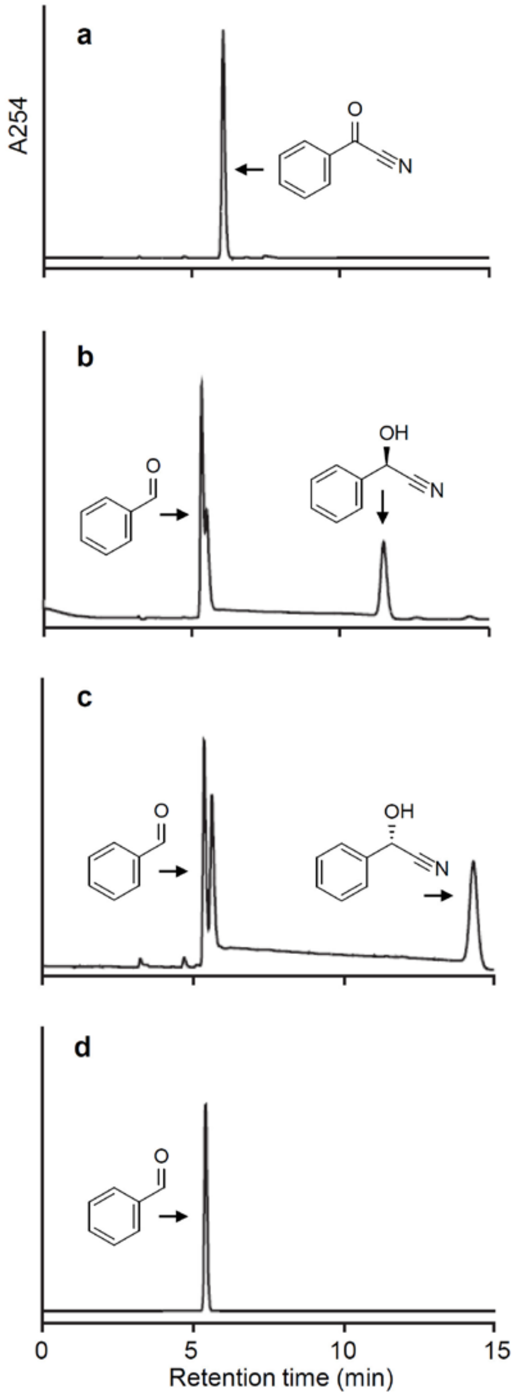

Fig. S2. Chromatograms of the authentic chemicals in the HPLC analysis. (a) Benzoyl cyanide. (b) (*R*)-mandelonitrile. (c) (*S*)-mandelonitrile. (d) Benzaldehyde. Because the absorbance of benzaldehyde is very strong, it is detected from the authentic (*R*)- and (*S*)-mandelonitrile in panels b and c, respectively.

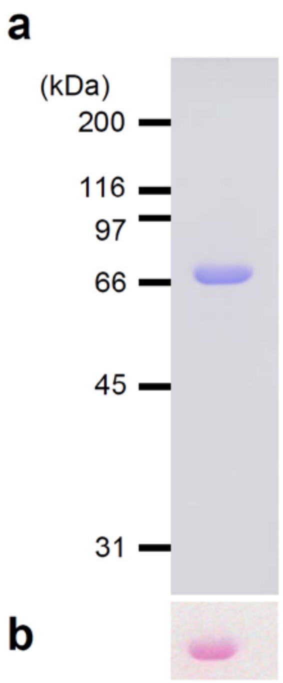

Fig. S3. (a) SDS-PAGE profile. ChuaMOX has a molecular mass of 67,000 Da. (b) PAS staining. ChuaMOX is a glycosylated enzyme.

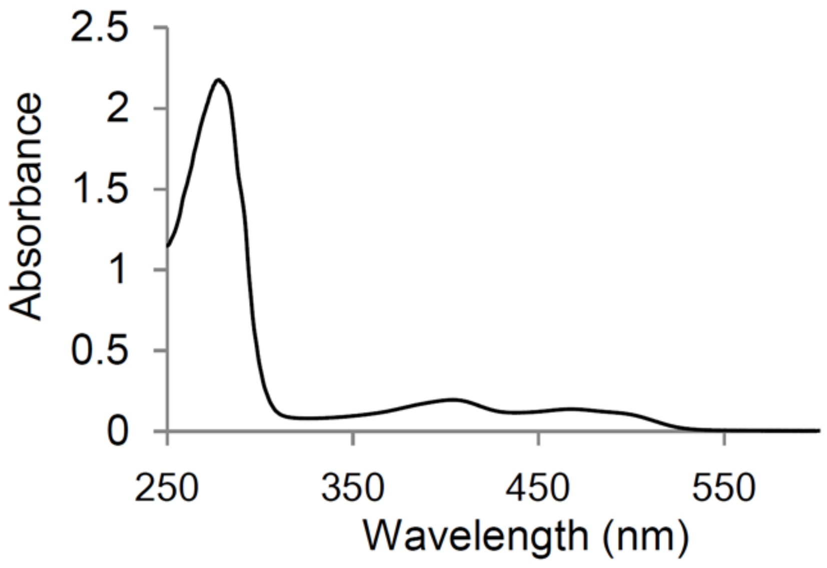

Fig. S4. UV-visible spectra. ChuaMOX shows two absorption maxima for the prosthetic group at 404 nm and 469 nm.

```
MGLLVLFVLA FVSVGHCQPK ETYDFIVVGA GSAGSVVANR LSELPNIKVL LLEAGGNETE  60
TSEVPLFAGQ LQLSPLDWNF TSTPQKNSCL AFWNQTCLWP QGKVLGGSSV LNYMIFVRGN 120
KKDFDDWAAL GNVGWDYNSV LPYFIKMENF TGPSTDAAIR GKTGPLTVGF VPYHTVLADT 180
FVSAGNENGY NTVDYNGHTQ TGVQRIQATT RDGQRCSTNK AYLWPIVHTR PNFVLKTHAT 240
VLKVLLNDKK AAIGVKYAIN GEEHTALASK EVILSAGALN SPQLLMLSGI GDPTDLQPFG 300
IKVLVENKGV GKNFQDHVAC GGVEWLIDQP VSLVTSRVVN DQTIKEWKDH GTGPLTIPSD 360
VEATAFVHTT TEYAAEDFPD IQLFYFSGTP ASDGGTGARY TTGFTNASWN GYYKEIENKD 420
AFSIYPVLLR PKSRGYIGLR SANPYDAPVI EPAYFTDPGR VDIDTMVRGV HVALNFGNSK 480
AFSKFGAKLH NATFPGCEPY PLHSDAYWEC LARHFISINF HPSSSCTMGD RTKTPLAVVD 540
NRLRVYGVKN LRVIDAAIIP LSPSGNTNGP TIMIGEKGSD LIKEDWKLKT PSG        593
```

Fig. S5. Deduced amino acid sequence of mandelonitrile oxidase from *C. hualienensis*. Amino acid sequences determined by protein sequencing are presented in red, and the signal peptide is shown in italics. The predicted flavin adenine dinucleotide binding motif is underlined, and *N*-glycosylation sites are indicated by arrowheads.

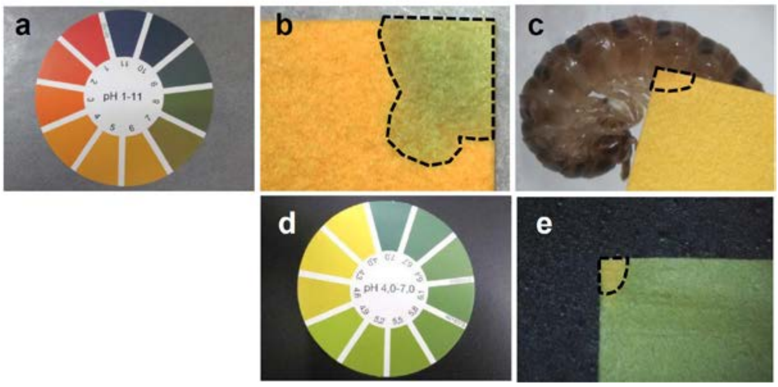

Fig. S6. Measurement of pH in the blood and the defense secretion. (a) Standard color chart for pH 1-11 indicator paper. (b) Blood. (c) Defensive secretion. (d) Standard color chart for pH 4-7 indicator paper. (e) Defensive secretion. The dashed line indicates the area of the dropped sample. The pH values of the blood and defensive secretion were 7 and 4.6, respectively.

Table S1. Purification summary

Procedure	Total protein (mg)	Total enzyme activity (U)	Specific activity (U/mg)	Purification fold	Recovery, %
100 animals	30,000	-	-	-	-
Crude extract from dissected bodies	54.0	151.0	2.8	1.00	100.0
DEAE Sepharose FF	4.61	98.7	21.4	7.64	65.4
Q Sepharose FF	0.634	53.3	84.1	30.0	35.3
Superdex 200 10/300 GL	0.161	21.1	131.0	46.8	14.0

Table S2. Thin layer chromatography analysis of prosthetic group of ChuaMOX

Prosthetic group	Relative mobility	
	Solvent 1	Solvent 2
Extract from boiled ChuaMOX	0.15	0.4
FAD	0.15	0.4
FMN	0.3	0.7

After separation with each solvent, the prosthetic group was detected under ultraviolet irradiation. Solvent 1, 1-butanol:acetic acid:water (3:1:1, v/v); solvent 2, 1-butanol:acetone:2-propanol:saturated boric acid (50:15:15:30, v/v).

Table S3. Stereoselectivity of ChuaMOX toward mandelonitrile

Substrate	Relative activity, %
(*R*)-Mandelonitrile	100.0
(*S*)-Mandelonitrile	2.4
Racemic mandelonitrile	49.5

ChuaMOX activity toward each test substrate (5 mM) was detected under standard assay condition using ATBS. The relative activity was expressed as a percentage of the enzyme toward (*R*)-mandelonitrile.

Table S4. Effects of chemicals and metals on ChuaMOX activity

Inhibitor	Relative activity, %	Metals	Relative activity, %
None	100.0	$MgCl_2$	100.0
Hydrazine	93.7	$MnCl_2$	78.3
Hydroxylamine hydrochloride	100.0	$CoCl_2$	100.0
Semicarbazide hydrochloride	92.0	$NiCl_2$	100.0
Pargyline	100.0	$CuCl_2$	100.0
Isoniazid	88.0	$ZnCl_2$	100.0
Iproniazid	80.0	$FeCl_3$	124.6
EDTA	100.0		
1,10-Phenanthroline	84.1		
2,2'-Bipyridyl	87.3		
8-Hydroxyquinoline	56.0		
Sodium azide	28.6		
Potassium cyanide	12.7		
N-Ethylmaleimide	96.8		
Iodoacetic acid	71.4		

Effects of chemicals and metals on ChuaMOX activity were detected under standard assay condition using ATBS by addition of 1 mM chemicals or metals at pH 5. Relative activity was expressed as a percentage of the enzyme without chemicals or metals.

Table S5. Test compounds

Racemic mandelonitrile, (*R*)-mandelonitrile, (*S*)-mandelonitrile, 2-hydroxy-2-(*o*-tolyl)acetonitrile, 2-hydroxy-2-(2-methoxyphenyl)acentonitrile, 2-hydroxy-2-(4-methoxyphenyl)acetonitrile, 2-(2-chlorophenyl)-2-hydroxyacetonitrile, 2-(2,6-dichlorophenyl)-2-hydroxyacetonitrile, 2-(3,5-dichlorophenyl)-2-hydroxyacetonitrile, 2-(2-bromophenyl)-2-hydroxyacetonitrile, 2-(3-bromophenyl)-2-hydroxyacetonitrile, 2-(3-bromophenyl)-2-hydroxyacetonitrile, 2-(4-bromophenyl)-2-hydroxyacetonitrile, 2-(2-aminophenyl)-2-hydroxyacetonitrile, benzyl alcohol, 1-phenylethanol, L-mandelic acid, 2-cyclohexyl-2-hydroxyacetonitrile, 2-hydroxy-3-phenylpropanenitrile, (*E*)-2-hydroxy-4-phenylbut-3-enenitrile, 3-cyclohexyl-2-hydroxypropanenitrile, 2-hydroxy-2-(naphthalene-1-yl)acetonitrile, 2-hydroxy-2-(naphthalene-2-yl)acetonitrile, 2-hydroxy-2-(quinolin-4-yl)acetonitrile, 2-([1.1'-biphenyl]-4-yl)-2-hydroxyacetonitrile, 2-propanol, 2-hydroxyheptanenitrile, (*E*)-2-hydroxyhept-3-enenitrile, (*E*)-2-hydroxyoct-3-enenitrile, (*E*)-2-hydroxynon-3-enenitrile, (3*E*,5*E*)-2-hydroxyocta-3,5-dienenitrile, (3*E*,5*E*)-2-hydroxyhepta-3,5-dienenitrile, (3*E*,5*E*)-2-hydroxydeca-3,5-dienenitrile, hydrazine, hydroxylamine hydrochloride, semicarbazide hydrochloride, pargyline, isoniazid, iproniazid, ethylenediamine-*N*,*N*,*N'*,*N'*-tetraacetic acid, disodium salt, dihydrate (EDTA·2Na), 1,10-phenanthroline, 2,2'-bipyridyl, 8-hydroxyquinoline, sodium azide, potassium cyanide, *N*-ethylmaleimide, iodoacetic acid, magnesium chloride, manganese chloride, cobalt chloride, nickel chloride, copper chloride, zinc chloride, and ferric chloride.

Table S6. Summary of analysis results of transcriptome sequencing

Total raw reads	729,477
Mean read length	446
Total clean reads	606,360
Total number of contigs	10,041
Mean contig size	987
Total number of singleton	26,938

Table S7. Primers

ChuaMOX-1, 5’-CATTGATACGATGGTTCGAGGCGTACACG-3’

ChuaMOX-2, 5’-CGGTTGTCAACCACAGCTAATGGAGTCTTG-3’

ChuaMOX-3, 5’-GGTTCGCATCCAGGGAATGTGGCATTGTGC-3’

ChuaMOX-4, 5’-CCCTTACACAGCGATGCTTATTGGGAATG-3’

P-ChuaMOX-5, P-5’-GTACAAGAGCTG-3’ (P indicates 5’ phosphorylated end.)

ChuaMOX-6, 5’-CAATGGACACACTCAAACAGGTGTACAACG-3’

ChuaMOX-7, 5’-CCATTGATGGCATACTTAACTCCAATAGCG-3’
